# Immune Checkpoint Inhibitors in Hepatocellular Carcinoma Before and After Liver Transplantation: A Systematic Review

**DOI:** 10.3390/cancers18081282

**Published:** 2026-04-17

**Authors:** Francesco Dituri, Livianna Carrieri, Maria Mosaico, Giusi Caragnano, Erica Villa

**Affiliations:** National Institute of Gastroenterology, IRCCS “S. De Bellis” Research Hospital, Via Turi 27, 70013 Castellana Grotte, Italy; francesco.dituri@irccsdebellis.it (F.D.); livianna.carrieri@irccsdebellis.it (L.C.); maria.mosaico@irccsdebellis.it (M.M.); giusi.caragnano@irccsdebellis.it (G.C.)

**Keywords:** hepatocellular carcinoma, liver transplantation, immune checkpoint inhibitors, washout interval, allograft rejection, graft loss, bridging therapy

## Abstract

Background/Objectives: Immune checkpoint inhibitors (ICIs) are increasingly used in hepatocellular carcinoma (HCC), but their application around liver transplantation (LT) remains controversial because checkpoint blockade may enhance antitumor immunity while disrupting graft tolerance. We systematically reviewed the available evidence on ICI exposure before LT and ICI therapy after LT for recurrent HCC. Methods: A PRISMA-guided systematic review with qualitative synthesis was performed. PubMed/MEDLINE, Embase, and Web of Science were searched from inception to 15 March 2026. Studies including adult patients with HCC treated with PD-1-, PD-L1-, and/or CTLA-4-targeting ICIs before LT or after LT for recurrent HCC were eligible. Results: Fifty-one studies were included. In the pre-LT setting, 25 studies reported 576 transplanted patients. Acute allograft rejection occurred in approximately 22% and graft loss in 3.8%, and shorter washout intervals were consistently associated with higher rejection risk. In the post-LT setting, 26 studies reported 117 recipients treated with ICIs; at least 22 rejection episodes (18.8%) were described, usually within 2–4 weeks of treatment initiation, with limited and inconsistent antitumor benefit. Conclusions: Pre-LT ICI use appears feasible in selected patients when adequate washout is respected. Post-LT ICI therapy remains high risk and should be reserved for highly selected cases within a multidisciplinary framework.

## Simple Summary

Immune checkpoint inhibitors (ICIs) are increasingly used in hepatocellular carcinoma (HCC), including in patients considered for liver transplantation or in those with tumor recurrence after transplantation. However, by activating antitumor immunity, these agents may also disrupt graft tolerance and trigger rejection. In this systematic review, pre-transplant ICI use appeared feasible in selected patients, especially when an adequate washout interval was respected before transplantation. In contrast, post-transplant ICI therapy was associated with a less favourable risk–benefit profile, with early rejection and limited or inconsistent oncologic benefit. These findings support a cautious, multidisciplinary approach and highlight the need for better biomarkers and prospective studies.

## 1. Introduction

Hepatocellular carcinoma (HCC) is one of the major global health burdens and remains one of the leading causes of cancer-related mortality worldwide. Its incidence continues to rise in parallel with chronic liver diseases such as viral hepatitis, alcohol-related liver disease, and metabolic dysfunction-associated steatotic liver disease [1,2]. Of curative therapy, liver transplantation (LT) has a unique capacity; it not only eliminates the tumour but also targets the cirrhotic substrate in which HCC occurs. Yet even after meticulous selection of the patients and various down-staging techniques, recurrence occurs in as much as 20% of patients [3,4].

In the last decade, immune checkpoint inhibitors (ICIs) have transformed the therapeutic landscape of advanced HCC. Agents targeting the programmed cell death-1 (PD-1) receptor, its ligand (PD-L1), and cytotoxic T-lymphocyte-associated antigen-4 (CTLA-4) have exhibited significant positive changes in survival rate in patients particularly in combination with anti-angiogenic treatment [5]. ICIs have rapidly become a standard systemic treatment option for advanced or unresectable HCC in appropriately selected patients, particularly those with preserved liver function and adequate performance status, although their positioning still varies across regional guidelines and treatment settings. Their use has also extended to broader clinical contexts, including selected patients undergoing locoregional therapies, whereas the most recent data in the adjuvant setting suggest caution regarding efficacy [6]. Aa a result, an increasing number of patients who are potential transplant candidates are now exposed to immunotherapy either intentionally (for downstaging or bridging) or incidentally as part of routine oncologic care.

The increasing use of ICIs has resulted in an immunological paradox with regard to liver transplantation. The liver is a highly tolerogenic organ with specific immune-regulatory pathways that facilitate peripheral tolerance to protect against excessive immune activation. The PD-1/PD-L1 pathway, which suppresses effector T-cell effects and is key to long-term graft acceptance, is central to this balance. Immune checkpoint blockade, by design, disrupts these inhibitory signals to restore antitumor immunity. In transplant recipients, however, the same mechanism may lower the threshold for alloimmune activation, culminating in acute and potentially irreversible graft rejection [7]. This dual properties—therapeutic benefit in cancer control versus immune-mediated graft injury—renders LT recipients uniquely vulnerable to ICI-related toxicity.

Two distinct and increasingly relevant scenarios may therefore occur. In the pre-transplant setting, ICIs have been explored as bridging or downstaging therapies in patients with tumor burden exceeding conventional transplant criteria with restoration of transplant eligibility. In the post-transplant setting, ICIs have been administered to treat recurrent HCC, a condition associated with poor prognosis and limited therapeutic options.

While both strategies are relevant and potentially lifesaving, they are associated with markedly different risk profiles. Reports of severe, early graft rejection following ICI exposure—particularly after transplantation—have raised substantial concern and led many transplant programs to regard ICIs as contraindicated in LT recipients [7].

The available evidence, however, is scarce and heterogeneous. Published data range from isolated case reports describing catastrophic graft failure to multicentre cohorts suggesting that transplantation after ICI exposure may be feasible under carefully controlled conditions. Key clinical questions remain unresolved, including the optimal timing of transplantation after ICI therapy, the relevance of washout intervals, the role of immunosuppression strategies, and the balance between oncologic benefit and graft-related risk. Importantly, no randomized trials address these issues, and guidance from international societies remains cautious and largely non-prescriptive.

In this context, there is an urgent need for an organized synthesis of the mounting clinical and mechanistic evidence surrounding immunotherapy use around LT in HCC. Specifically, a comparison between pre- and post-transplant ICI exposure-related adverse events might contribute to separating modifiable from non-modifiable risk factors and clarify whether a safe therapeutic window exists. The combination of such clinical observations with recent knowledge of the immune tolerance of the liver and of checkpoint biology may be conducive to making intelligent decisions in this population at elevated risk. We summarize the evidence reviewed for ICI exposure in HCC patients undergoing liver transplantation, based on pre- and post-transplant data. We contrast rejection incidence, timing, severity, and oncologic outcomes in these two scenarios, delineate parameters associated with safety and efficacy, and summarize important gaps in our understanding. This systematic revision aims to complement clinical impact with immunobiological knowledge to support an evidence-based framework for judicious and cautious use of immunotherapy in the liver transplant setting.

## 2. Materials and Methods

### 2.1. Study Design

**Figure 1:**
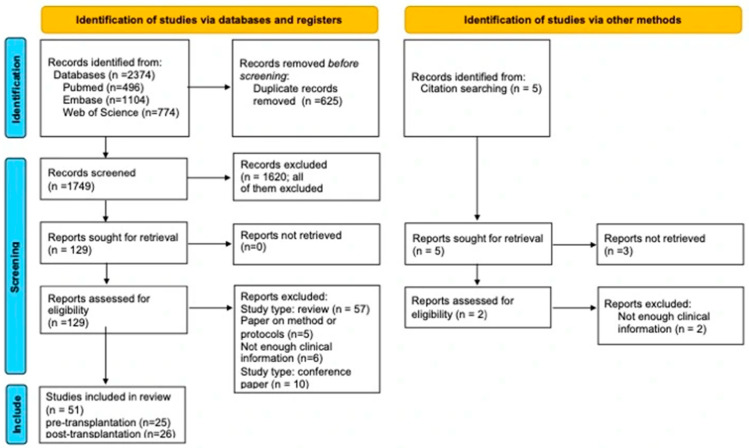
PRISMA flow diagram of study selection—identification of studies via databases and registers. The diagram illustrates the identification, screening, eligibility assessment, and inclusion of studies evaluating immune checkpoint inhibitor exposure in patients with hepatocellular carcinoma undergoing liver transplantation, in accordance with the PRISMA 2020 guidelines.

This work was conducted as a systematic review with qualitative synthesis, following the Preferred Reporting Items for Systematic Reviews and Meta-Analyses (PRISMA) guidelines. The PRISMA checklists are provided as Supplementary Table S1. The study selection process is shown in Figure 1. The review protocol was registered in PROSPERO (CRD420261350175). Given the heterogeneity of study designs, patient populations, ICI regimens, timing of exposure relative to LT, outcome definitions, and reporting completeness, and for the possibility of partial overlap between some multicentre cohorts and earlier single-centre reports, a formal quantitative meta-analysis was not planned. Even within apparently similar subsets, pooling was considered likely to generate estimates with misleading precision; therefore, where numerical summaries are presented, they should be interpreted as descriptive and hypothesis-generating rather than causal.

### 2.2. Literature Search Strategy

A systematic literature search was performed in PubMed/MEDLINE, Embase, and Web of Science for studies published from database inception to 15 March 2026. The search strategy combined controlled vocabulary and free-text terms related to hepatocellular carcinoma, liver transplantation, and immune checkpoint inhibitors. The full database-specific search strategy is provided in Supplementary Table S2. Reference lists of relevant articles and reviews were also screened manually to identify additional eligible studies. Because this is a rapidly evolving field, the search end date was prespecified to ensure transparency and reproducibility.

### 2.3. Eligibility Criteria

Studies were eligible if they included adult patients with hepatocellular carcinoma treated with PD-1-, PD-L1-, and/or CTLA-4-targeting immune checkpoint inhibitors, either before LT (bridging or downstaging setting) or after LT for recurrent HCC. Case reports, case series, and retrospective or prospective observational studies were included, provided that they reported at least one relevant outcome, including allograft rejection, graft loss, tumor response, recurrence, or survival. Studies focusing on non-HCC malignancies, non-checkpoint-based immunotherapies, preclinical or animal models, conference abstracts without full text, or non-English publications were excluded.

### 2.4. Study Selection and Data Extraction

Two reviewers independently screened titles and abstracts for eligibility. Full texts of potentially relevant studies were then assessed for inclusion. Disagreements were resolved by consensus.

Data were independently extracted by two reviewers using a standardized form. Extracted variables included study characteristics, number of patients, timing of ICI exposure relative to transplantation, immunotherapy regimen, washout interval (for pre-LT exposure), immunosuppressive therapy, rejection events, graft loss, survival, and oncologic outcomes. Missing or incompletely reported data were recorded as not reported (NR). Potential overlap across multicentre cohorts and earlier single-centre reports was specifically assessed during full-text review and data extraction by comparing institution, recruitment period, clinical setting, and cohort composition; where overlap was suspected, the most comprehensive dataset was prioritized for quantitative counting, whereas smaller reports were retained only when they appeared non-overlapping or provided unique mechanistic or pathologic information. The study selection process is illustrated in the PRISMA flow diagram (Figure 1).

### 2.5. Assessment of Study Quality

Given that most included studies were case reports, case series, or small observational cohorts, no formal risk-of-bias tool was applied, as instruments such as ROBINS-I or Newcastle–Ottawa are not readily applicable in a consistent manner across predominantly descriptive evidence. Study quality was instead assessed qualitatively based on the clarity of patient selection, temporal relationship between ICI exposure and transplantation, completeness of outcome reporting, and availability of histologic confirmation of rejection. These considerations were considered during data synthesis and interpretation, and greater interpretive weight was assigned to studies with clearer patient selection, more complete outcome reporting, and biopsy-confirmed rejection. No formal certainty-of-evidence framework was applied. Confidence in the body of evidence was instead evaluated qualitatively, given the predominance of case reports, case series, and small retrospective cohorts, as well as the substantial clinical and methodological heterogeneity across studies.

### 2.6. Data Synthesis

Because of substantial clinical and methodological heterogeneity, the results were synthesized qualitatively. Studies were grouped according to the timing of ICI exposure relative to transplantation (pre-LT vs. post-LT), and outcomes were summarized descriptively in tabular and narrative form. No formal assessment of reporting bias or certainty of evidence was undertaken; instead, these issues were considered qualitatively in light of the predominance of retrospective and descriptive reports, the likely publication bias toward severe or unusual events, and the inconsistent reporting of key transplant-related variables. Regimen-specific differences were summarized narratively, but no formal subgroup analysis by ICI class was attempted because treatment type was strongly confounded by timing of exposure, indication, combination therapy, and transplant context.

### 2.7. Ethics Statement

This study was based exclusively on published studies and did not involve primary data collection. Therefore, ethical approval was not required.

## 3. Results

The process described in the Preferred Reporting Items for Systematic Reviews and Meta-Analyses (PRISMA) framework and the following flowchart are presented (Figure 1). The search and screen identified studies reporting immune checkpoint inhibitor (ICI) exposure in patients with HCC undergoing LT, either before transplantation or after transplantation for recurrent disease. The systematic search identified 2374 records through database searching (PubMed/MEDLINE, *n* = 496; Embase, *n* = 1104; Web of Science, *n* = 774). After removal of 625 duplicate records, 1749 records remained for title and abstract screening. Of these, 1720 were excluded. The remaining 129 reports were assessed in full text for eligibility, of which 77 were excluded (57 reviews, 5 methodological/protocol papers, 6 reports with insufficient clinical information, and 10 conference papers). Ultimately, 51 studies were included in the qualitative synthesis (25 before transplantation; 26 after transplantation). The final dataset consisted of case reports, case series, and observational cohort studies, as no randomized controlled trials have been reported in this clinical context.

Because of heterogeneity in study design, immunotherapy regimens, timing of exposure relative to LT, and outcome reporting, results were synthesized qualitatively, as specified in the Methods Section. Results are presented based on ICI exposure relative to transplantation. Outcomes associated with pre-LT ICI exposure are reported first, emphasizing transplant feasibility, washout periods, incidence and timing of rejection. Results from post-LT ICI therapy in recurrent HCC are then summarized, particularly focusing on graft-related outcomes and antitumor efficacy.

### 3.1. Immune Checkpoint Inhibitor Exposure Before Liver Transplantation

A total of 25 studies reporting pre-LT exposure to ICIs in patients with HCC were included, encompassing 576 transplanted patients treated with ICIs prior to LT (Table 1) [8,9,10,11,12,13,14,15,16,17,18,19,20,21,22,23,24,25,26,27,28,29,30,31]. Study designs included case reports, single-centre series, and large multicentre retrospective cohorts.

Pre-LT ICI therapy was administered primarily with bridging or downstaging intent in patients with tumor burden exceeding conventional transplant criteria or at high risk of wait-list dropout. Most patients received anti-PD-1 or anti-PD-L1 monotherapy, although combination regimens, including atezolizumab plus bevacizumab and PD-1 plus CTLA-4 blockade, were reported in more recent series. The number of ICI cycles and integration with locoregional therapies varied widely across studies, reflecting the absence of standardized neoadjuvant protocols.

Across the studies included in Table 1, acute allograft rejection after LT occurred in approximately 22% of transplanted patients following pre-LT ICI exposure. Graft loss attributable to rejection was uncommon, occurring in 11 patients (3.8%). Reported washout intervals ranged from 7 to 229 days, with consistently higher rejection rates observed when transplantation occurred within 30 days of the last ICI dose. When biopsy-confirmed, rejection was predominantly T-cell-mediated, consistent with rapid reactivation of alloimmune responses following checkpoint blockade.

Importantly, rejection risk appeared strongly influenced by the interval between the last ICI dose and transplantation. In a large multicentre cohort, Guo et al. [14] demonstrated that a washout interval exceeding 30 days was associated with a significantly lower risk of allograft rejection, a finding subsequently confirmed in a large international cohort reported by Moeckli et al. [9]. In the latter study, the overall rejection rate ranged from 20% to 28% but declined to <10% when the washout interval exceeded approximately 50 days. By contrast, ultra-short washout intervals (<2 weeks) were associated with severe early graft injury or rejection, including fatal outcomes in early case reports [30]. We therefore interpreted the available evidence as indicating a time-dependent reduction in risk rather than a universally safe threshold; despite heterogeneity in reported washout durations, intervals between 30 and 50 days repeatedly identified a lower-risk, but not risk-free, window across studies.

Although rejection was relatively frequent, irreversible graft failure remained uncommon. At least 4 deaths and 4 retransplantations were explicitly reported; in addition, 6 further graft losses were reported in the largest cohort but were not disaggregated into death versus retransplantation. These severe outcomes were predominantly associated with very short washout intervals or highly immunogenic PD-1 monotherapy exposure.

Immunosuppressive regimens, both for induction and maintenance, were heterogeneous across studies. In reports providing protocol-level detail, maintenance therapy most often consisted of calcineurin inhibitor-based regimens, frequently combined with mycophenolate mofetil and corticosteroids. Basiliximab or anti-thymocyte globulin induction were reported less often. No specific immunosuppressive strategy consistently mitigated rejection risk in patients transplanted shortly after ICI exposure, underscoring the dominant role of pre-existing immune activation. At the same time, these observations must be interpreted cautiously because the apparent effect of immunosuppressive regimen is confounded by baseline disease severity, urgency of transplantation, center-specific practice, and the timing of ICI discontinuation.

#### 3.1.1. Pathological and Biomarker Assessment

Systematic assessment of PD-1/PD-L1 expression in graft tissue was largely absent. In at least one large cohort, such analyses were explicitly not performed, precluding correlations between intragraft immune phenotype and rejection risk. This paucity of biomarker-driven stratification substantially limits translational interpretation and highlights the need for studies integrating graft tissue profiling with clinical outcome data.

#### 3.1.2. Oncologic Outcomes

The oncologic outcomes of transplanted patients were generally favourable, with high rates of successful downstaging to transplant eligibility and low early post-LT recurrence rates during short- to mid-term follow-up. Importantly, extended washout intervals were not associated with increased post-LT recurrence, suggesting that delaying transplantation to reduce rejection risk does not appear to compromise oncologic control [8,10].

Overall, ICI exposure in HCC before LT was associated with feasible transplantation in carefully selected patients, with a moderate descriptive risk of rejection and a low risk of graft loss. In the current evidence base, “selected” refers pragmatically to patients managed in experienced multidisciplinary transplant programs, with a meaningful oncologic indication for bridging or downstaging, disease control under ICI-based therapy, no uncontrolled extrahepatic disease or severe immune-related toxicity, and the possibility of observing an adequate washout interval before transplantation. More than the immunosuppressive regimen itself, the ICI washout interval emerged as the most consistent observable determinant of rejection, although this association remains vulnerable to confounding. Collectively, these findings indicate that successful transplantation after ICI exposure is achievable, provided that an adequate washout period is observed. Recent systematic reviews and individual patient–level meta-analyses published in 2024–2025 substantiate the conclusions of the present review [33,34]. Across independent cohorts, rejection risk after pre-LT ICI exposure consistently correlates with shorter washout intervals, while no consistent signal has emerged implicating a specific checkpoint inhibitor or maintenance immunosuppressive strategy. These pooled analyses further suggest that deferring transplantation to allow adequate immune reset does not adversely impact oncologic outcomes, reinforcing the feasibility of a time-adapted approach. Together, emerging aggregated data provide converging evidence that pre-LT ICI therapy can be integrated into transplant pathways under carefully controlled temporal conditions, whereas post-LT use remains considerably less predictable. In this respect, the recent expert consensus from the Italian Association for the Study of the Liver (AISF) aligns with the available evidence, emphasizing that although pre-LT ICI exposure is associated with a meaningful risk of acute rejection, the likelihood of irreversible graft failure remains comparatively low and appears to be strongly modulated by adequate treatment discontinuation before transplantation [35].

### 3.2. Immune Checkpoint Inhibitor Therapy After Liver Transplantation for Recurrent HCC

A total of 26 studies reporting post-LT exposure to ICIs were included (Table 2) [7,36,37,38,39,40,41,42,43,44,45,46,47,48,49,50,51,52,53,54,55,56,57,58,59,60]. Most reports concerned recurrent HCC, although some more recent cohorts also included de novo malignancies or mixed recurrent primary liver tumours. Overall, 117 LT recipients were treated with ICIs after transplantation. The evidence base remained dominated by case reports and small series, but more recent data also included a multicenter retrospective cohort of 52 recipients and a prospective pilot cohort of 18 recipients. Anti-PD-1-based therapy predominated, whereas atezolizumab plus bevacizumab represented the most frequently reported PD-L1-based regimen in recent series.

The interval between LT and ICI initiation was highly variable, ranging from 9.5 months to more than 11 years. Across the studies included in Table 2, at least 22 allograft rejection episodes were reported among 117 treated recipients (18.8%), although precise pooling remains limited by heterogeneous populations and incomplete event reporting in a minority of studies. When reported, rejection was predominantly acute T-cell-mediated and usually occurred early, most often within the first 2–4 weeks after ICI initiation.

In the largest cohort [35], rejection occurred in 7/52 recipients (13%) at a median of 27 days and was histologically confirmed in 6/7 cases; in addition, early severe cellular rejection with graft failure was documented in several case reports [53]. Although the prominence of early severe events may partly reflect publication bias, the temporal pattern of rejection within days to a few weeks was also observed in larger cohorts and therefore appears biologically plausible rather than being confined to isolated reports.

Importantly, the accumulating evidence no longer supports the assumption that atezolizumab-based regimens are uniformly rejection-free. Although no rejection was reported in individual cases or small series [38,39,40,42,44,46,47,50,54,57], rejection events were also described in newer atezolizumab-containing retrospective cohorts, indicating that PD-L1-based strategies may attenuate but do not eliminate alloimmune risk. Available data further suggest that proactive intensification of immunosuppression and continued calcineurin inhibitor exposure at ICI initiation may mitigate rejection risk, whereas shorter time from LT and recent immunosuppression minimization may increase vulnerability. Although mTOR-based regimens were associated with an apparent lower incidence of rejection in isolated reports [39,40], the number of cases is very limited and, importantly, these patients were typically very late after transplantation (≥9 years), a context inherently associated with lower alloimmune risk during immune checkpoint inhibitor therapy. Overall, available post-LT evidence remains insufficient to implicate any specific maintenance immunosuppression regimen as a consistent determinant of ICI-related rejection. Similarly, no reliable subgroup analysis by ICI class was possible, because PD-1-, PD-L1-, and CTLA-4-based regimens were used in small and clinically non-comparable populations, often in combination with anti-angiogenic agents or other systemic therapies. Collectively, these data indicate that time from transplantation and the underlying immunologic context outweigh immunosuppression type or ICI class as observable determinants of rejection risk.

#### 3.2.1. Histologic Picture

Pre-treatment liver graft biopsies with immune profiling before immune checkpoint inhibitor therapy remain rarely reported in post-transplant hepatocellular carcinoma. In a prospective pilot study restricted here to the 18 HCC cases, biopsy-proven rejection occurred in 3 patients (16.7%) despite uniformly negative baseline graft PD-L1 staining [36]. Earlier case-level reports likewise suggested that graft tissue assessment is feasible, but such observations remain isolated. By contrast, in most published reports, liver biopsies were obtained only after graft dysfunction to document rejection, limiting the discovery of predictive histologic or immunophenotypic biomarkers. These data support further evaluation of protocolized pre-ICI graft biopsy integrated with transcriptomic and peripheral immune profiling to identify alloimmune risk states and guide biologically informed patient selection.

#### 3.2.2. Antitumor Activity and Survival Outcomes

Antitumor efficacy was heterogeneous and generally limited. Most case reports described stable disease or progression, whereas cohort-level data suggested only modest survival benefit in selected subgroups. The absence of uniform response criteria, short follow-up in many reports, and the scarce number of consistent cohorts preclude firm conclusions on this issue. Importantly, the overall clinical value of post-LT ICI therapy is constrained not only by graft-related risk, but also by the generally poor and inconsistent oncologic payoff observed across studies.

Overall, post-transplant ICI therapy remains a high-risk strategy, characterized by a meaningful early risk of predominantly T-cell-mediated rejection, limited and inconsistent oncologic benefit, and the absence of validated biomarkers to guide patient selection. It should therefore be considered exceptional and reserved for highly selected patients after careful multidisciplinary discussion of graft-related risks, expected oncologic benefit, time from transplantation, and baseline immunologic context.

## 4. Conclusions

ICIs have reshaped the therapeutic landscape of hepatocellular carcinoma, yet their integration into the LT setting remains inherently complex and high risk.

Current evidence supports a conditional and time-dependent role for ICIs before transplantation, where carefully selected patients may benefit from bridging or downstaging strategies provided that an adequate washout interval is respected to mitigate rejection risk. In practical terms, such patients are presently those managed in experienced multidisciplinary centers, with controlled tumor burden under therapy, no uncontrolled extrahepatic disease or severe immune-related toxicity, and the possibility of delaying LT long enough to allow a lower-risk washout interval. In contrast, post-transplant ICI therapy for recurrent HCC is associated with unpredictable and potentially life-threatening allograft rejection, limited and inconsistent antitumor efficacy, and a lack of validated biomarkers to guide patient selection.

Notably, combination regimens incorporating anti-angiogenic agents may modulate immune activation and have shown signals of improved disease control in small post-transplant series, but their impact on graft tolerance remains insufficiently defined. Across settings, timing relative to transplantation and the underlying immunologic context emerge as more influential determinants of outcome than the specific immunosuppressive regimen or ICI agent. Current evidence supports a time-dependent reduction in pre-LT rejection risk with longer washout, but not a universally safe minimum threshold; transplantation within <30 days of the last ICI dose appears higher risk and should be avoided whenever possible, whereas longer intervals, particularly beyond approximately 50 days in the largest cohorts, appear associated with lower risk. Until prospective data and biologically informed risk stratification tools become available, the use of ICIs in liver transplant recipients should remain exceptional, confined to highly selected cases within a multidisciplinary framework and accompanied by transparent discussion of graft-related risks.

## Figures and Tables

**Table 1:** Immune checkpoint inhibitor exposure before liver transplantation in patients with hepatocellular carcinoma: detailed extraction of immunosuppression, rejection phenotype, and timing.

Study	Design/Setting	N	ICI Regimen(s)	Washout (last ICI → LT)	Maintenance IS	Acute Rejection/Graft Loss	Median Time to Rejection	Key Oncologic Notes/Outcomes
Bhoori [8]	Prospective phase II (single centre)	16	Atezolizumab + bevacizumab	Median 57.5 days (IQR 29–87)	Steroid boluses for rejection; intensified early CNI; no mTOR switch	4 (25%) 1 death; 2 re-transplantations *	Early post-LT	2 yr RFS 90%; 2 yr OS 94%; 1 recurrence (6.2%)
Moeckli [9]	International retrospective cohort focused on washout safety	119	Multiple anti-PD-1/PD-L1 agents	Modelled categories: <30 d, 30–50 d, >50 d	CNI-based ± MMF ± steroids; no significant differences between groups	24/119 (20.2%) rejection; washout <30 d and 30–50 d strongly associated with rejection; graft loss due to rejection in 6	Median 9 days	Key practical conclusion: washout >50 days appears “safe” re rejection risk; longer washout not linked to worse RFS at ~36 months.
Tabrizian [10]	Prospective, multicentre Western experience (7 centres)	17	Atezolizumab + Bevacizumab	Stopped ≥4 weeks pre-LT	NR	2 mild biopsy-proven rejections, no severe rejection, no graft loss	NR	ORR 94%; downstaging to Milan 82%; pathologic response 88%; 1 & 3 yr OS 94.2% & 88.2%.
Zhang & Zhang [11]	Case report	2	Camrelizumab + sorafenib (Case 1); Tislelizumab + Lenvatinib (Case 2)	Case 2: 62 days; Case 1: prolonged interval	Tacrolimus + MMF + steroids	0 (1 late liver failure unrelated to rejection)	-	Pathologic CR in both
Xu [12]	Multicentre retrospective study	25	ICIs (heterogeneous)	exact distribution unknown	Tacrolimus ± MMF ± Sirolimus	3/25 (12%)	POD 2-124	Local recurrence:12 (48%) Deaths: 4 (16%) (1 due to TCMR)
Lv [13]	Prospective randomized pilot trial (PLENTY)	10 (ICI arm)	Pembrolizumab + Lenvatinib	Median 60.5 days (range 25–193)	Methylprednisolone taper; tacrolimus + MMF; sirolimus from 6–8 weeks	0	-	12 mo RFS 70%; 30 mo tumor-specific RFS 37.5%
Guo [14]	Multicentre retrospective cohort	83	Heterogeneous pre-LT ICIs	≥30 days protective	CNIs, mTOR, antiproliferative agents and corticosteroids	23/83 (27.7%) allograft rejection; TLAT ≥ 30 d protective	11.0 (7.0–25.0) days posttransplant.	Rejection independently associated with worse OS (HR ~ 9.96) during short follow-up
Kulkarni [15]	Retrospective single-centre case series	5	Atezolizumab + Bevacizumab	Median 89 days (range 38–114)	Tacrolimus + MMF + steroids induction; everolimus added at 30 days post-LT	0	N/A	No HCC recurrence during median 10-month follow-up; no rejection; wound-healing complications in 3/5
Abdelrahim [16]	Retrospective single-centre cohort	6	Atezolizumab/bevacizumab (n = 4); nivolumab (n = 1); nivolumab/ipilimumab (n = 1)	Median 5 months (range 1–41 months)	Tacrolimus + mycophenolate (all); 1 patient also received prednisone; 1 everolimus added	0	N/A	Successful downstaging in all; 0 clinical rejection; recurrence in ≥1 patient during follow-up
Lu [17]	Retrospective cohort	39	Pretransplant ICI exposure (agents NR)	NR	NR	9/39 (23.1%) acute rejection 5/39 (12.8%) rejection-related mortality	POD 6–16	In recipients with MVI, pretransplant ICI exposure appeared protective for overall survival; RFS/OS in ICI-exposed MVI patients were comparable to non-ICI recipients without MVI
Fang [18]	Multicentre retrospective cohort study	209	Pre-LT ICIs (heterogeneous)	NR	Heterogenous	36 (17.2%)	10 days after LT	Predictive model may help stratify high-risk patients for post-LT rejection
Wang [19]	Retrospective single-centre cohort	16	PD-1 inhibitors (nivolumab n = 2; pembrolizumab n = 7; sintilimab n = 4; camrelizumab n = 2; mixed sequence n = 1)	Median 21 days (rejection group) vs. 60 days (non-rejection)	Basiliximab induction; tacrolimus + MMF ± sirolimus	9/16 (56.3%)	7 days	1-year recurrence 25%; no immune-related graft loss
Ohm [20]	Case series (3 non-consecutive cases)	3	Atezo/Beva or Ipi/Nivo	2–229 days		0 rejections reported: good graft function at follow-up	-	Provides case-level washout granularity and mixed regimens (incl. Ipi/Nivo).
Chouik [21]	Case report	1	Atezolizumab + Bevacizumab	30 days	Steroids, tacrolimus, mycophenolate mofetil	No rejection	-	Alive
Aby [22]	Case report	1	Nivolumab	16	Mycophenolate mofetil, tacrolimus, steroids	Rejection High dose corticosteroids	Day 3	Alive
Schnickel [23]	Single-centre case series	5	Nivolumab	0.3–11 months	Steroids + tacrolimus + MMF; rATG in severe cases	2 rejections; 1 retransplantation	<3 months	No HCC recurrence
Kang [24]	Case report (adolescent)	1	Pembrolizumab (3 cycles)	138 days	Tacrolimus + sirolimus	0	N/A	No recurrence at 48 months
Dave [25]	Single-centre retrospective waitlist/transplant cohort	5	Nivolumab	Median 105 days (range 11–354)	NR; ATG used in 3 ICI-treated recipients	2/5 (40%) biopsy-proven acute rejection; 2/5 graft loss *	NR	No waitlist dropout in ICI group vs. 10.4% in non-ICI group; favourable explant features vs. non-ICI; rejection occurred only when last ICI dose was <90 days before LT
Qiao [26]	Single-centre cohort	7	PD-1 inhibitors	Washout at 42 days		BPAR 14.3% (1/7; mild, treated)	NR	Demonstrated feasibility with standardized washout; includes peri-op immune monitoring. (Frontiers)
Dehghan [27]	Case report	1	Nivolumab	35 days	Steroid, tacrolimus, mycophenolate mofetil	Acute rejection	10 days	Retrasplantation
Lizaola-Mayo [28]	Case report	1	Nivolumab/ Ipilimumab	63 days	NR	No rejection	-	Alive
								
Sogbe [29]	Case report	1	Durvalumab	90 days	NR	No rejection	-	Alive
Chen [30]	Case report	1	Toripalimab (10 cycles)	93 days	Tacrolimus + steroids	Fatal acute hepatic necrosis (immune-mediated) (death)	<72 h	Not evaluable
Nordness [31]	Case report	1	Nivolumab	8 days	Tacrolimus, mycophenolate mofetil, steroids	Severe/fatal early graft injury consistent with ICI-associated rejection/necrosis	6 days	Landmark “high-risk” case that shaped caution about very short washout
Schwacha-Eipper [32]	Case report	1	Nivolumab	105 days	NR	No rejection	-	No recurrence, alive

* Patients described as alive in the “Key oncologic notes/outcomes” column were alive at the end of follow-up as reported in the original publication. Abbreviations: Beva: Bevacizumab anti-VEGF; CNI: calcineurine inhibitors; ICI, immune checkpoint inhibitor; Ipi: ipilimumab; IS: Immunosuppression; HCC, hepatocellular carcinoma; LT, liver transplantation; MMF, mycophenolate mofetil; NR, not reported; PD-1, programmed cell death-1; PD-L1, programmed death-ligand 1; OS, overall survival; Nivo: Nivolumab (anti-PD-1); Nivo/Beva, nivolumab plus bevacizumab; N/A, not applicable; NR: not reported; POD: post-operative days; TCMR: T-cell mediated rejection.

**Table 2:** Immune checkpoint inhibitor exposure after liver transplantation in patients with recurrent hepatocellular carcinoma.

Study	Design/Setting	n	ICI Regimen	Time from LT → ICI	Maintenance Immunosuppression (Reported)	Rejection (Confirmation & Phenotype)	Time to Rejection	PD-1/PD-L1 Staining in Graft	Antitumor Outcome
De Martin [36]	Retrospective multi-center	52	Anti-PD1 (Nivolumab 31%); anti-PD-L1 (mainly atezolizumab + bevacizumab) 69%; HCC-recurrence patients treated with anti-PD-L1 + bevacizumab	Median 4.5 years/IQR 2.6–9.9)	At ICI initiation: corticosteroids 58%, CNI 83%, mTORi 52%, MMF 33%; IS proactively increased in 65%	7/52 (13%); histologically proven in 6/7; moderate/severe TCMR (Banff 7–8), no AMR features; plasma cell–rich component in some cases	Median 27 days (IQR 23–57)	NR	HCC recurrence indication in 32/52 (62%); overall survival after ICI at 3, 6, 12 months: 65%, 46.9%, 38.9%; rejection associated with markedly worse survival
He [37]	Prospective single-center, open-label, single-arm pilot trial	18	Toripalimab 240 mg q3w; concomitant targeted therapy in many patients	NR; median recurrence-free survival after LT before study entry 405 days	NR in detail; baseline post-LT immunosuppression continued	3/18 (16.7%) biopsy-proven acute rejection (BPAR); Banff RAI 4–8; severe in 1 case	After cycle 1 in 2 patients; after cycle 3 in 1 patient	All graft biopsies PD-L1 negative before ICI; post-treatment PD-L1 positivity detected in 16/18	ORR 0%; median post-recurrence survival 24.6 months; 1-year OS 55%, 2-year OS 24%
Yao [38]	Retrospective single center	4	Atezolizumab	NR	Tacrolimus-based (protocol not fully detailed)	Rejection events reported (details limited)	NR	1/4	Progressive disease
Giudicelli [39]	Retrospective cohort	7	Atezolizumab + bevacizumab	11–29 months	Tacrolimus/steroids; MMF	1 rejection (14%)	Immediately after first dose	NR	5/7 progressive disease and death
Di Marco [7]	Retrospective single center	5	Nivolumab (1) or nivolumab + bevacizumab (4)	Median ~14.5 mo (4–106)	Tacrolimus ± MMF ± steroids	1/5 moderate–severe rejection (biopsy-proven TCMR); steroid-responsive	~2 weeks	NR	Mixed; cohort-level OS signal favouring nivo + beva vs. regorafenib
Zhou [40]	Case report with immune profiling	1	Atezolizumab + bevacizumab	~11 years	Sirolimus + MMF + low-dose prednisone	No rejection	N/A	Graft PD-L1 negative	Progressive disease
Rudolph [41]	Case series	2	Atezolizumab + bevacizumab	9.5 mo; 24 mo	Tacrolimus + MMF + prednisone	No rejection	N/A	Tumor PD-L1 negative (1 case)	Progressive disease
Yang [42]	Preliminary report	1	Atezolizumab + bevacizumab	NR	Tacrolimus-based (details limited)	No rejection	N/A	NR	Disease control reported; short follow-up
Jin [43] *	Case report	1	Low dose nivolumab 40 mg ×2, combined with lenvatinib 8 mg/day	~2 months post-LT	Initially tacrolimus + MMF; changed to everolimus + MMF before nivolumab	No rejection reported; liver function remained normal throughout treatment	N/A	Graft PD-L1: NR; explant/tumor PD-L1 negative	No radiologic recurrence and no recurrence/metastasis during 2-year follow-up
Dai [44]	Case report	2	Camrelizumab ± targeted therapy	NR	Low-dose tacrolimus + sirolimus + MMF during ICI	No rejection	N/A	PD-L1 negative (tumor & graft)	Stable disease for 3 months in one case and 10 months in the other, followed by progression and death in 1
Shi [45]	Case series	6	Toripalimab	11–17 months		1 rejection		1/6 PD-L1 expression +	Stable disease in 1, 2 progressions, 2 not evaluable, and 1 death following rejection
Ben Khaled [46]	Case report	1	atezolizumab/bevacizumab	4 years	NR	No rejection	-	NR	Initial stabilization then progression and death after 10 months
Al Jarroudi [47]	Case series	3	Nivolumab (2); Pembrolizumab (1)	NR	Tacrolimus-based (details limited)	2/3 rejection (cellular, biopsy in ≥1 case)	Early (<2 wks)	NR	Poor oncologic outcomes overall
Qiu [48]	Case report	1	Camrelizumab	4.3 years	Sirolimus	No rejection	N/A	NR	After cycle 13: progressive disease, new liver metastases
Amjad [49]	Case report	1	Nivolumab	~2 years	Tacrolimus-based	No rejection	N/A	NR	Successful treatment of disseminated HCC
Anugwom & Leventhal [50]	Case report	1	Nivolumab	NR	Tacrolimus-based	Immune-mediated cholestatic hepatitis (no clear TCMR)	NR	NR	ICI discontinued; illustrates immune toxicity distinct from rejection
Zhuang [51]	Case report	1	PD-1 inhibitor	~1 year	Tacrolimus-based	Acute cellular rejection (biopsy-proven)	~10 days	NR	Progressive disease
Pandey & Cohen [52]	Case report	1	Ipilimumab (CTLA-4 inhibitor)	~8 years	Low-dose tacrolimus (stable)	No rejection	N/A	NR	Durable response, long-term disease control
Rammohan [53]	Case report (LDLT)	1	Pembrolizumab	~2 years	Tacrolimus-based	Acute graft rejection (clinical)	NR	NR	Progressive disease; fatal outcome reported
Gassmann [54]	Case report	1	Nivolumab (single dose)	~2 years	Tacrolimus (re-intensified during rejection)	Severe biopsy-proven TCMR	~1 week	NR	Fatal graft failure
DeLeon [55]	Case report	1	Nivolumab	~3 years	Tacrolimus + steroids	Acute cellular rejection (biopsy-proven)	<2 weeks	NR	Progressive disease
Tio [56]	Case report	1	Nivolumab	~8 years	Tacrolimus	No rejection	N/A	NR	Partial response
Friend [57]	Case report	2	Nivolumab	Late post-LT	NR	Acute cellular rejection	NR	NR	Death
De Toni & Gerbes [58]	Case report	1	Nivolumab	~8 years	Tacrolimus (tapered during ICI)	No rejection	N/A	NR	Sustained nivolumab treatment with preserved graft function
Varkaris [59]	Case report	1	PD-1 pathway inhibitor	~2 years	Tacrolimus (stable)	No rejection	N/A	NR	Preserved graft function; disease control
Gomez [60]	Case report	1	Nivolumab	~2 years	NR	Acute cellular rejection	After the 2nd dose	NR	NR

* Preemptive ICI with biochemical response with AFP/AFP-L3 normalization: (nivolumab started after marker rise on POD45 and 2 weeks of lenvatinib), Abbreviations: Beva: Bevacizumab; Camr: Camrelizumab (anti-PD-1); ICI, immune checkpoint inhibitor; IS: Immunosupression; HCC, hepatocellular carcinoma; LT, liver transplantation; MMF, mycophenolate mofetil; NR, not reported; PD-1, programmed cell death-1; PD-L1, programmed death-ligand 1; OS, overall survival; Nivo: Nivolumab (anti-PD-1); Nivo/Beva, nivolumab plus bevacizumab; N/A, not applicable, NR: not reported; POD: post-operative days.

## Data Availability

No new data were created or analysed in this study.

## Supplementary Materials

The following supporting information can be downloaded at: https://www.mdpi.com/article/10.3390/cancers18081282/s1, Table S1. presents the full electronic search strategies used for this systematic review. Table S2. PRISMA 2020 Checklist [61].
