# Supplementary material for: Immune Checkpoint Inhibitors in Hepatocellular Carcinoma Before and After Liver Transplantation: A Systematic Review
Source: Cancers (Basel). 2026 Apr 17;18(8):1282. doi: 10.3390/cancers18081282 (PMC13114636; doi:10.3390/cancers18081282)
Supplement: Supplementary file 1 [file cancers-18-01282-s001.zip › Supplementary Table S1 .pdf]

**Supplementary Table S1.**

Supplementary Table S1 presents the full electronic search strategies used for this systematic review. Searches were conducted in PubMed/MEDLINE, Embase, and Web of Science from database inception to 15 March 2026. The strategies combined terms related to hepatocellular carcinoma, liver transplantation, and immune checkpoint inhibitors. Reference lists of included studies and relevant reviews were also screened manually to identify additional eligible reports.

**Databases Searched**

- PubMed/MEDLINE
- Embase
- Web of Science Core Collection

**Search Period**

From database inception to 15 March 2026

---

**1. PubMed / MEDLINE**

("Carcinoma, Hepatocellular"[Mesh] OR "hepatocellular carcinoma" OR HCC)
AND
("Liver Transplantation"[Mesh] OR "liver transplant*" OR "hepatic transplant*")
AND
( "Immune Checkpoint Inhibitors"[Mesh]
  OR immunotherap*
  OR "immune checkpoint inhibitor*"
  OR "checkpoint inhibitor*"
  OR "PD-1"
  OR "PD-L1"
  OR "CTLA-4"
  OR nivolumab
  OR pembrolizumab
  OR atezolizumab
  OR durvalumab
  OR tremelimumab
  OR ipilimumab
  OR camrelizumab)

**Filters applied:**

- Language: English
- Species: Humans

---

**2. Embase**

('hepatocellular carcinoma'/exp OR 'hepatocellular carcinoma' OR hcc)
AND
('liver transplantation'/exp OR 'liver transplant*' OR 'hepatic transplant*')
AND
( 'immune checkpoint inhibitor'/exp
  OR immunotherap*
  OR 'immune checkpoint inhibitor*'
  OR 'checkpoint inhibitor*'
  OR 'programmed cell death 1'
  OR 'programmed death ligand 1'
  OR 'cytotoxic t lymphocyte associated protein 4'

OR nivolumab
OR pembrolizumab
OR atezolizumab
OR durvalumab
OR tremelimumab
OR ipilimumab
OR camrelizumab)

**Limits:**

- Humans
- English language

---

### 3. Web of Science Core Collection

("hepatocellular carcinoma" OR HCC)
AND
("liver transplantation" OR "liver transplant*" OR "hepatic transplant*")
AND
(immunotherap*
OR "immune checkpoint inhibitor*"
OR "checkpoint inhibitor*"
OR "PD-1"
OR "PD-L1"
OR "CTLA-4"
OR nivolumab
OR pembrolizumab
OR atezolizumab
OR durvalumab
OR tremelimumab
OR ipilimumab
OR camrelizumab)

**Indexes searched:**

- SCI-EXPANDED
- SSCI

**Language:** English

---

### 4. Additional Search Methods

- Manual screening of reference lists from included articles and relevant reviews
- Citation tracking of key studies

---

### Notes for Reviewers

- No date restrictions were applied.
- Both pre- and post-liver transplantation ICI exposure were included.
- Case reports, case series, and observational studies were eligible due to the rarity of the clinical scenario.
